# The multistep progression of areca nut-induced oral cancer: a mechanistic roadmap from pathogenesis to precision therapy

**DOI:** 10.1093/lifemedi/lnag011

**Published:** 2026-03-27

**Authors:** Na Yu, Wenqiu Cai, Congyi Zhang, Qiyue Cai, Zisong Zhang, Yuqing Hu, Yan Sun, Kaiyao Yin, Feng Ren, KangXin Chang, MeiLing Jin, Dongxia Li, Liwen Zhang, Heming Wu, Mengwei Li

**Affiliations:** School of Life Science and Technology, China Pharmaceutical University, Nanjing 210009, China; School of Life Science and Technology, China Pharmaceutical University, Nanjing 210009, China; School of Life Science and Technology, China Pharmaceutical University, Nanjing 210009, China; School of Life Science and Technology, China Pharmaceutical University, Nanjing 210009, China; School of Basic Medicine and Clinical Pharmacy, China Pharmaceutical University, Nanjing 210009, China; School of Health Sciences, The University of Manchester, Manchester M13 9PL, United Kingdom; Mudi Meng Honors College, China Pharmaceutical University, Nanjing 210009, China; Mudi Meng Honors College, China Pharmaceutical University, Nanjing 210009, China; School of Life Science and Technology, China Pharmaceutical University, Nanjing 210009, China; School of Life Science and Technology, China Pharmaceutical University, Nanjing 210009, China; School of Life Science and Technology, China Pharmaceutical University, Nanjing 210009, China; School of Life Science and Technology, China Pharmaceutical University, Nanjing 210009, China; School of Life Science and Technology, China Pharmaceutical University, Nanjing 210009, China; School of Life Science and Technology, China Pharmaceutical University, Nanjing 210009, China; Department of Oral and Maxillofacial Surgery, Affiliated Stomatological Hospital of Nanjing Medical University, Nanjing 210000, China; School of Life Science and Technology, China Pharmaceutical University, Nanjing 210009, China

**Keywords:** oral cancer, areca nut, oral submucous fibrosis, immune microenvironment reprogramming, targeted therapy

## Abstract

Areca nut is classified as a Group 1 carcinogen by the International Agency for Research on Cancer. It is a widely consumed psychoactive substance with profound cultural roots in regions including Hunan, Hainan, and Taiwan of China. Its key bioactive components include alkaloids (e.g. arecoline and arecaidine) and areca nut-specific nitrosamines, that induce DNA damage, reactive oxygen species bursts, and chronic inflammation in oral tissues. Coupled with mechanical trauma from chewing, these insults drive the malignant progression of oral submucous fibrosis to oral cavity carcinomas. This review systematically outlines the pathological progression from normal oral mucosa to invasive oral cavity carcinomas, highlighting two core mediators of oral submucous fibrosis carcinogenesis: immune microenvironment reprogramming and oncogenic signaling activation. Furthermore, this review elaborates the molecular mechanisms of areca nut-induced oral cancer, providing a theoretical foundation for biomarker discovery and the development of novel therapeutic strategies. It also provides actionable guidance for reducing the incidence of areca nut-related oral cavity carcinomas and improving patient prognosis.

## Introduction

Oral cavity carcinomas (OCCs) are epithelium-derived malignancies arising from oral mucosal linings, initiated by dysregulated proliferation and differentiation of keratinocytes. Characterized by invasive growth and metastatic potential, these tumors infiltrate adjacent tissues and pose severe threats to patient health, representing the 16th most incident malignancy worldwide [1]. Oral cancer etiology is multifactorial, involving viral infections [e.g. high-risk human papilloma virus (HPV)], lifestyle factors (smoking, alcohol, areca nut chewing), chronic physical/chemical irritation, and nutritional deficiencies (e.g. vitamins A, C, E) [2]. OCCs affect multiple subsites including the tongue, gingiva, buccal mucosa, palate, and floor of mouth [3].

Oral squamous cell carcinoma (OSCC) accounts for over 90% of oral cancers. In 2020, global new diagnoses exceeded 370,000 with over 170,000 deaths [4]. Notably, over 30% of OSCC cases occur in South and Southeast Asia—regions with the highest oral cancer mortality risk—strongly associated with areca quid consumption [5]. In China, Hunan Province represents a core areca nut consumption region, where the prevalence of areca nut chewing among adult residents exceeds 40% in certain local jurisdictions. High chewing prevalence is also documented in major production regions including Hainan and Taiwan. This geographically widespread chewing behavior constitutes a key risk factor, which contributes to the significantly higher incidence of oral cancer in these areas relative to the national average [6, 7]. Epidemiological surveys further demonstrate that the chewing prevalence among males in Taiwan, China is ∼50%; notably, the population attributable fraction of areca nut chewing for oral cancer in Taiwan, China reaches as high as 53.7% [8].

Areca nut (*Areca catechu* L.) serves as both traditional herbal medicine and stimulant, typically chewed with lime for central nervous system effects. Its composition includes carbohydrates (26%–47%), polyphenols (11%–26%), fats (1.3%–17%), alkaloids (0.15%–0.67%), and tannins [9]. Alkaloids (e.g. arecoline) and polyphenols are the primary bioactive components. Despite reported digestive and antidepressant properties, areca nut carries documented risks including carcinogenicity. Arecoline, the most abundant alkaloid, undergoes enzymatic hydrolysis to arecaidine and induces reactive oxygen species (ROS) production in epithelial cells, triggering cell cycle arrest, DNA damage, and cytotoxicity [10, 11]. Based on compelling evidence, International Agency for Research on Cancer classified areca nut as Group 1 carcinogen in 2003.

Oral squamous cell carcinoma impairs basic oral functions. It causes dysphagia, speech disorders, and pain—all of which affect nutrition and social interaction. Additionally, this tumor is highly invasive. It can metastasize to the cervical lymph nodes, lungs, and liver. In severe cases, it may lead to multiple organ failure [12]. Treatments including surgery, radiotherapy, and chemotherapy often cause facial disfigurement and functional impairment, inducing psychological trauma and diminished quality of life. The diagnostic and therapeutic processes also impose substantial economic burdens on families and healthcare systems [13].

**Figure 1.**
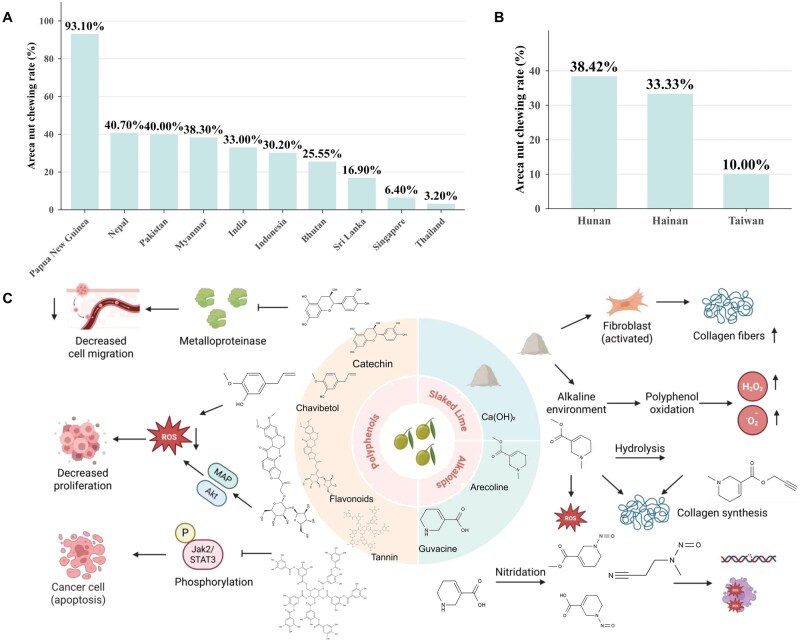
Areca-nut chewing prevalence and key carcinogens. (A) Prevalence of areca nut chewing in major areca-consuming regions of Asia. The highest prevalence is observed in Papua New Guinea (93.10%), followed by Nepal (40.70%), Pakistan (40.00%), Myanmar (38.30%), India (33.00%), Indonesia (30.20%), Bhutan (25.55%), Sri Lanka (16.90%), Singapore (6.40%), and Thailand (3.20%). (B) Prevalence of areca nut chewing in major areca-consuming regions of China. The prevalence is 38.42% in Hunan, 33.33% in Hainan, and 10.00% in the Taiwan region of China. (C) Cancer-related components of areca nut. Key carcinogens include alkaloids, slaked lime, and polyphenols. Arecoline (hydrolyzed to arecaidine) stimulates collagen synthesis and induces inflammation/ROS. Guvacine-derived nitrosamines cause DNA breaks and oxidative stress. Slaked lime promotes fibroblast changes and collagen formation, while its alkalinity oxidizes polyphenols to ROS that damage cells. Polyphenols exhibit protective effects: catechin inhibits matrix metalloproteinases (MMPs); chavibetol scavenges ROS; flavonoids downregulate ROS and suppress proliferation via Akt/MAPK; tannins induce apoptosis via Jak2/STAT3 inhibition. Created by BioRender and Datawrapper.

This review systematically elucidates key molecular mechanisms of areca quid-induced oral carcinogenesis, particularly oral submucous fibrosis (OSF) malignant transformation to OSCC. It emphasizes how bioactive components (alkaloids and areca-specific nitrosamines) trigger DNA damage, oxidative stress, and chronic inflammation. These factors, combined with mechanical friction, drive immune microenvironment remodeling and critical pathway activation, promoting OSF–OSCC progression. By integrating current research advances, this work addresses knowledge gaps in areca quid carcinogenicity and provides a theoretical framework for developing preventive and therapeutic strategies (Fig. 1).

## Pathological characteristics and mechanisms of areca nut-induced oral cancer

### Characteristics and progression of areca nut-associated precancerous lesions

Normal oral mucosa is the structural foundation for maintaining oral physiological functions. Dysregulation of cell differentiation in normal oral mucosa induces two key changes including abnormal epithelial proliferation and differentiation defects. Ultimately, these changes lead to the development of various precancerous lesions. This stage constitutes the initial phase of OSCC development. Oral epithelial dysplasia (OED) is one of the primary precancerous lesions of OSCC [11]. In clinical practice, inter-observer diagnostic consistency is poor. This is especially true for differentiated dysplasia, where lesions are dominated by structural disorders with inconspicuous cellular atypia. Such discrepancies can affect the assessment of OED progression and subsequent clinical management [14, 15].

**Figure 2.**
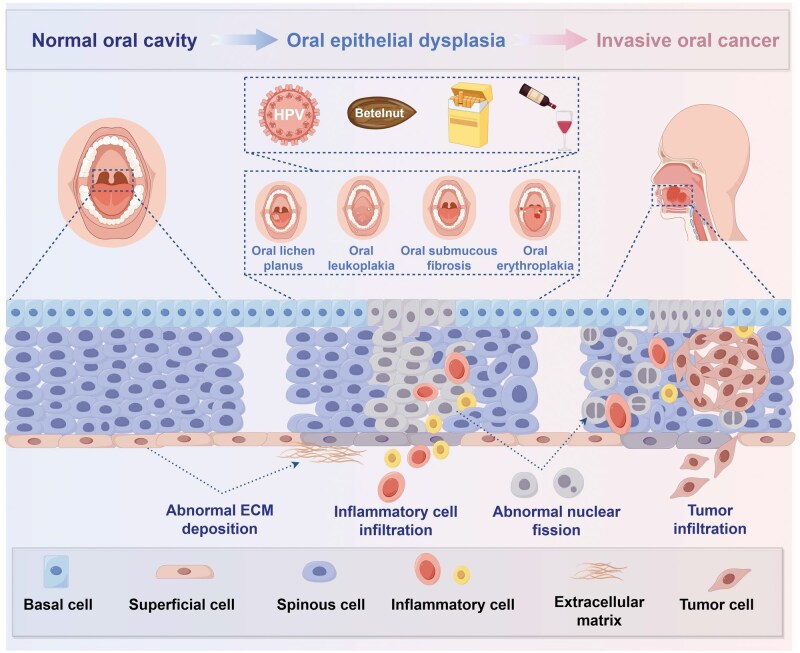
Core role of areca nut-induced immune microenvironment in OSF-to-OSCC malignant transformation. ANE promotes oral fibrosis and carcinogenesis via EMT process, regulating miRNA expression, myofibroblast activation, and collagen deposition to form pathological fibrosis favoring OSCC development. Areca nut-induced immune dysregulation is manifested as promoting lymphocyte apoptosis, inhibiting neutrophil function, reducing Th1 cytokine production, increasing MDSC expansion, suppressing T-cell activation and IFN-γ secretion, and elevating pro-carcinogenic inflammatory factors, which collectively driving tumor immune escape. The spatially heterogeneous TME features CAF1/CAF2-immune cell interactions through specific ligand–receptor pairs, hindering immune infiltration and promoting escape.

In regions where areca nut chewing is common, oral leukoplakia (OL) and OSF are the most frequent subtypes of oral potentially malignant disorders. Areca nut chewing is the primary etiological factor for both lesions [16], and carries a significant risk of malignant transformation [17]. Their pathogenesis is closely linked to the chemical stimulation and mechanical injury induced by areca nut (Fig.  2).

#### Oral leukoplakia

According to WHO clinical diagnostic criteria, OL is defined as a non-removable white plaque or patch on the oral mucosa, excluding other clinically or histopathologically defined lesions. The chemical carcinogens in areca nut (e.g. arecoline) and mechanical friction from its fibers act synergistically. They induce abnormal proliferation of oral mucosal epithelial cells, leading to pathological hyperkeratosis or epithelial dysplasia. This eventually forms ill-defined white lesions. Pathologically, OL is divided into two subtypes: “homogeneous OL,” appears as uniform white plaques with a smooth surface; “non-homogeneous OL,” associated with erythema, ulceration, or nodular changes and carries a significantly higher risk of malignant transformation. Existing studies have shown substantial heterogeneity in the malignant transformation rate of OL (0.1%–36.4%). This variation is attributed to lesion subtype, follow-up duration, and geographical differences [18].

#### Oral submucous fibrosis

OSF is a chronic, progressive precancerous lesion. It is characterized by dysregulated collagen metabolism in the lamina propria and submucosa of the oral mucosa. The hallmark pathological feature is abnormal collagen deposition in the extracellular matrix (ECM) [19]. This is accompanied by epithelial atrophy, basal cell hyperplasia, collagen hyalinization, and abrupt keratinization transitions. These pathological changes suggest that clonal genetic alterations are the key drivers of malignant transformation [16, 17, 20]. Clinically, OSF progresses in stages [21]: “in the early stage,” patients mainly experience sensory abnormalities (e.g. burning, pain, taste disturbance), worsened by irritating foods. “The progressive stage” is marked by three cardinal signs: pale and hardened mucosa, palpable fibrous bands, and progressive trismus. In severe cases, the interincisal distance may be less than 2 cm, seriously impairing chewing and speech. “The advanced stage” is characterized by restricted tongue movement and complete loss of mucosal elasticity. Long-term areca nut chewing subjects the oral mucosa to persistent mechanical abrasion and friction from areca nut fibers. A cohort study in Taiwan, China, involving over 1 million patients with oral precancerous lesions demonstrated that OSF patients have a 10% malignant transformation rate, significantly higher than that of the general population [17].

**Figure 3.**
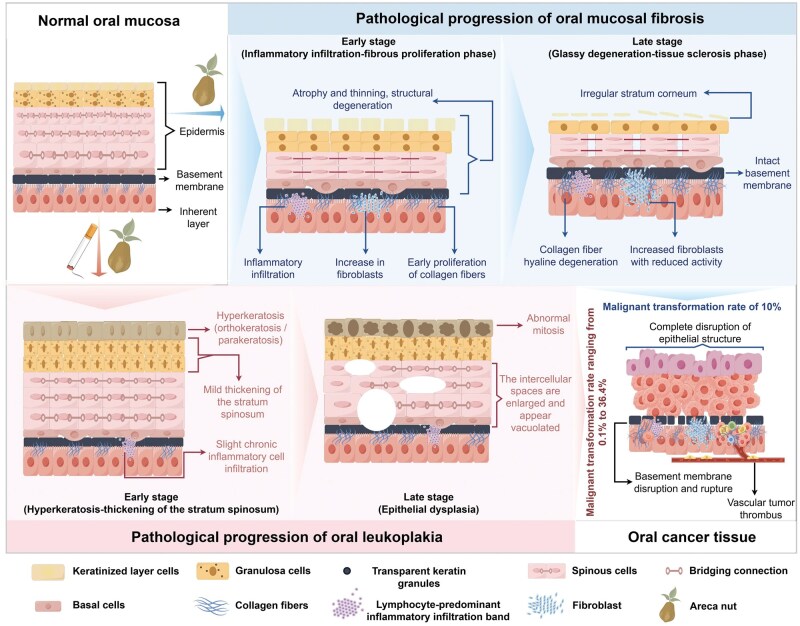
Schematic diagram of the pathological progression of oral mucosa from healthy state to malignant lesions. On the left side of the figure is the normal oral mucosa, which exhibits a clear layered structure of “epithelial layer–basement membrane–lamina propria,” with regular epithelial ridges and orderly arranged collagen fibers. The upper-middle section illustrates the two-stage progression of OSF: the early stage is characterized by inflammatory infiltration and collagen proliferation, while the late stage is marked by collagen hyaline degeneration, vascular stenosis, and epithelial atrophy, with the basement membrane remaining intact. The lower-middle section depicts the pathological progression of OL: the early stage presents epithelial hyperkeratosis and acanthosis, and the late stage progresses to epithelial dysplasia (accompanied by cellular polarity disorder and nuclear atypia). On the right side is the OSCC tissue, which shows malignant features such as complete destruction of the epithelial structure, basement membrane disruption, tumor cell infiltration, and vascular tumor thrombus formation.

As precancerous lesions progress, cellular atypia and invasiveness gradually increase, ultimately leading to basement membrane disruption and the development of invasive and metastatic OSCC, marking the transition to the tumor invasion and metastasis stage. The prognosis of OSCC patients is closely linked to tumor histological grade and invasive pattern [16, 20]: moderately differentiated tumors are the most common (67.2%), well-differentiated tumors exhibit keratin pearls, and poorly differentiated tumors display marked cellular atypia and high mitotic activity. OSF has a cervical lymph node metastasis rate of up to 41.1%, predominantly involving levels I–III (corresponding to N1–N2b stages) and frequently accompanied by extracapsular extension [16, 17]. Aggressive invasive patterns (worst pattern of invasion (WPOI) grades 4–5), invasion depth > 5 mm, and bone invasion are independent predictors of lymph node metastasis. Primary tumor location also influences metastatic potential: tongue cancer (36.2%) and floor-of-mouth cancer (21.0%) have significantly higher metastasis rates due to the abundant lymphatic drainage in these regions [19, 20] (Fig. 3).

### Major mechanisms underlying areca nut-induced oral precancerous lesions and progression to carcinoma

Oral squamous cell carcinoma is a multifactorial, multistage malignancy driven by gene mutation accumulation, chronic inflammatory microenvironment, epigenetic dysregulation, immune escape, and environmental exposures (e.g. areca nut, tobacco). These factors collectively induce the malignant transformation of oral mucosal epithelial cells from precancerous lesions to invasive carcinoma.

A core mechanism involves gene mutation accumulation and genomic instability: activated proto-oncogenes (e.g. EGFR, STAT3) and inactivated tumor suppressor genes disrupt the proliferation-apoptosis balance, triggering malignancy [22–24]. Tumor suppressor gene mutations further aggravate genomic instability (76%–80% of OSCC cases carry TP53 mutations [25]; CDKN2A and PIK3CA are other frequently mutated genes [26]).

Chronic inflammation and immune dysregulation also serve as key drivers: metabolically activated benzo[a]pyrene in tobacco causes DNA damage and TP53 mutations [27]; alcohol-derived acetaldehyde impairs DNA repair and induces oxidative stress [28]; E6/E7 proteins of high-risk HPV inactivate p53/pRb, and HPV-associated OSCC is characterized by p16 overexpression and wild-type TP53 [29, 30]. Recent studies show that long-term exposure to areca nut/tobacco or high-risk HPV infection fosters an immunosuppressive tumor microenvironment (TME) in the oral cavity, which promotes tumor immune escape and metastasis via three pathways: regulatory T cells (Tregs) secrete inhibitory cytokines [31]; M2-type tumor-associated macrophages facilitate angiogenesis, areca nut extract (ANE) induces their polarization, and their infiltration correlates with prognosis [32–40]; the circadian gene *PER2* downregulates PD-L1 expression [41–44]. Thus, clarifying the differential features of core mechanisms under distinct inducing factors is critical for the clinical diagnosis and treatment of oral cancer subtypes.

#### Areca nut mediates multi-level gene–protein networks to drive carcinogenesis

The process by which areca nut induces OSF and ultimately progresses to carcinoma involves dysregulation of multi-layered gene regulatory networks.

At the non-coding RNA level, areca nut specifically modulates the expression of various carcinogenesis-related microRNAs (miRNAs) [45]. For instance, it upregulates miR-497 to activate the transforming growth factor (TGF)β1/Smads axis, thereby driving myofibroblast activation and collagen deposition [46]; exosomal miR-17-5p enhances TGF-β-mediated fibrotic progression via dual inhibition of Smad7/WWP1 [47]; whereas epigenetic silencing of miR-34a abrogates its inhibitory effect on the Axl/Akt/GSK-3β pathway, this impairs both cell cycle regulation and the capacity to suppress epithelial–mesenchymal transition (EMT), and restoring miR-34a expression has been shown to reduce xenograft tumor volume by 65% [48]. Furthermore, downregulation of miR-200b/c can promote ZEB1/ZEB2-mediated EMT and confer anti-apoptotic properties [49, 50]. Concurrently, arecoline induces aberrant expression of carcinogenic long non-coding RNAs (lncRNAs): LUCAT1 triggers DNA repair defects and chemotherapy resistance through the NRF-2-mediated oxidative stress pathway [49]; MIR31HG activates the Wnt5A/MAPK pathway to promote cell proliferation and metastasis [51]; UCA1 functions as a competitive endogenous RNA (ceRNA) to disrupt cell junction structures and activates the Wnt/Notch pathway, driving EMT [51]; HIF1A-AS1 directly mediates fibroblast activation and the fibroblast-to-cancer transition [52].

At the gene mutation level, areca nut-induced OSCC exhibits high-frequency *TP53* mutations (70%–80%), which lead to functional inactivation of the p53 protein and impair cell cycle arrest and apoptosis capabilities [53]. Arecoline also suppress the expression of mismatch repair genes (*MLH1* and *MSH2*), resulting in mismatch repair deficiency, which in turn drives mutation accumulation and is associated with poor prognosis. Mutations in the autophagy-related gene *ATG2A* and the apoptosis gene *CASP8* are mutually exclusive, suggesting a dominant role for the autophagy pathway in areca nut-associated carcinogenesis [54]. Additionally, HRAS activating mutations can constitutively activate the MAPK/ERK pathway, promoting abnormal cell proliferation [55].

Regarding genomic instability, areca nut alkaloids directly induce DNA double-strand breaks, leading to the enrichment of short insertions/deletions (indels), an increase in structural variation breakpoints, and exhibit characteristic mutational signatures (e.g. Signature 1/5) [56, 57]. Unlike HPV-associated oral cancer characterized by E6-mediated p53 degradation, high CDKN2A expression, and chromosome 3q amplification, areca nut-associated oral cancer possesses unique molecular markers, including high-frequency *TP53* mutations, low CDKN2A expression, and abundant indels and structural variations [24].

At the protein level, bioactive components of areca nut (e.g. arecoline and its nitrosamines derivatives) drive OSCC and OSF through dysregulation of protein expression. Intracellular glutathione (GSH) accumulation induces oxidative stress, which subsequently upregulates the expression of HSP27, HSP47 (inhibitable by *N*-acetylcysteine), metallothionein-1, and heme oxygenase-1 [52, 58]. Activation of the COX-2/PGE2 signaling pathway (inhibitable by NS-398) regulates the expression of HSP27 and HSP47 [52, 58, 59]. The PI3K/AKT and ERK/MAPK pathways upregulate the expression levels of HSP47 and β-catenin [59, 60]; activation of the NOTCH signaling pathway induces the expression of NOTCH1, its downstream target HES1, and the cadherin FAT1 [60]. ANE also upregulates amphiregulin to activate the EGFR/ERK signaling pathway and induce EMT [61], and depends on copper ions to activate lysyl oxidase (LOX), thereby promoting collagen cross-linking [62, 63]. Furthermore, upregulation of keratin 17 enhances cell migration and invasion [62]; S100A4 promotes OSF progression by regulating TIMP1 and MMP9 [59]; and insulin-like growth factor-1 and Cystatin C contribute to fibrosis formation by promoting ECM deposition [64, 65].

In summary, areca nut promotes oral carcinogenesis and fibrosis through the combined effects of oxidative stress, synergistic activation of multiple signaling pathways, and dysregulation of protein networks.

#### Areca nut triggers the oxidative stress–inflammation–tissue damage malignant transformation cascade

Under normal physiological conditions, the oxidative system (e.g. ROS, RNS) and antioxidant system (e.g. GSH, SOD, CAT) in oral mucosal cells maintain a dynamic balance, ensuring cellular homeostasis. Chewing areca nut, however, induces oxidative stress and chronic inflammation, forming a vicious cycle of the “oxidative stress–inflammation axis”: ROS from alkaloid metabolism causes DNA damage and inflammatory factor release, which further aggravates oxidative stress. Prolonged exposure impairs compensatory mechanisms (e.g. SIRT3/HO-1), leading to carcinogenic effects. Concurrently, areca nut chewing significantly elevates local oral pH (> 8), accelerating the oxidation of phenolic compounds and substantial ROS release [66]. Areca nut alkaloids interact with copper ions (Cu^2+^), further increasing ROS via mitochondrial enzymes (e.g. cytochrome P450) and NOX-1/NOX-4, while inhibiting antioxidants like SOD. Excess ROS directly attacks DNA, forming 8-hydroxy-2'-deoxyguanosine (8-OHdG) adducts and inducing DNA damage [67]. Furthermore, ROS activates the MAPK/ERK pathway, promoting NF-κB nuclear translocation and initiating transcription and secretion of pro-inflammatory factors, such as interleukin-6 (IL-6) and TNF-α [67]. Although IL-6 and TNF-α aid wound healing by promoting keratinocyte proliferation, sustained ROS impedes healing and causes recurrent ulcers. ROS also downregulates mTOR, weakening its anti-cancer function [67]. Notably, the JNK (c-Jun N-terminal kinase) inhibitor SP600125 upregulates arecoline-induced IL-6 transcription, suggesting impaired DNA repair and compensatory inflammation, though this requires further validation [67].

The cellular impact of ANE exposure is time-dependent, spanning two stages: short-term protection and long-term damage. Short-term ANE exposure induces intracellular ROS, which upregulates SIRT3. SIRT3 promotes nuclear translocation and activation of Foxo3a via deacetylation. Activated Foxo3a induces SOD2 expression, clearing excess ROS and maintaining short-term oxidative balance [68]. During long-term exposure, however, sustained ROS exceeds cellular clearance capacity, leading to ROS accumulation. This not only causes DNA damage and cell cycle arrest but also suppresses SIRT3 expression, impairing Foxo3a activation and antioxidant defense, thereby promoting carcinogenesis [68]. Additionally, ANE depletes intracellular GSH, inducing compensatory HO-1 overexpression. Short-term HO-1 activation clears ROS via bilirubin production, but prolonged GSH depletion shifts HO-1 from a protective factor to a pro-tumor survival factor. Tobacco smoke components (e.g. benzopyrene) synergize with ANE to amplify HO-1 induction [69], worsening oxidative damage and carcinogenic risk.

**Figure 4.**
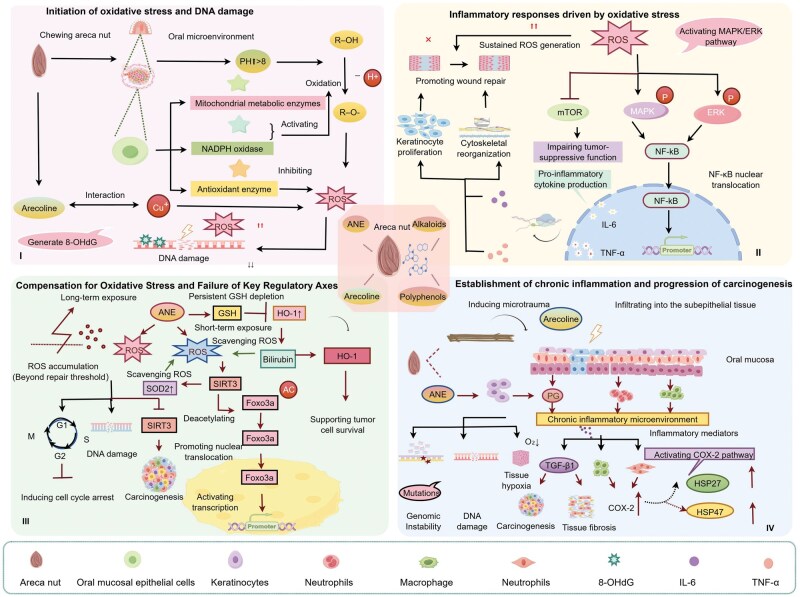
Core vicious cycle of areca nut promoting carcinogenesis through oxidative stress-inflammation axis. Chewing areca nut triggers ROS burst through pH elevation and arecoline-Cu^2+^, directly causing DNA damage. ROS activates MAPK/ERK–NF-κB pathway to induce pro-inflammatory factors IL-6/TNF-α release, and inflammatory microenvironment exacerbates oxidative stress forming vicious cycle. Short-term exposure activates compensatory SIRT3–Foxo3a/SOD2 and HO-1 antioxidant axes; chronic exposure causes ROS accumulation exceeding repair threshold → SIRT3 inactivation + HO-1 pro-carcinogenic shift (tobacco-synergized). Coarse fiber-induced microtrauma promotes inflammatory infiltration; TGF-β1-driven fibrosis; persistent COX-2/HSP expression collectively promote genomic instability and carcinogenesis.

Beyond chemical components, the coarse fibrous structure of areca nut causes repeated mucosal friction during chewing, mechanically damaging epithelial cells. Damaged cells release ROS and initiate a stress response, but persistent friction sustains oxidative stress. Microtraumas allow ANE components to infiltrate submucosal tissue, recruiting macrophages and neutrophils and forming a chronic inflammatory microenvironment [70]. ANE also stimulates keratinocytes to secrete prostaglandins, exacerbating inflammation. TGF-β1 produced at inflammatory sites promotes fibrosis and carcinogenesis [11], while persistent inflammation causes tissue hypoxia, DNA damage, and genomic instability (Fig. 4). Inflammatory factors stimulate COX-2 overexpression in macrophages and endothelial cells. COX-2 serves as both a downstream effector of oxidative stress and a mediator that amplifies it, linking “oxidative stress” and “inflammatory response.” Clinical studies confirm increased inflammatory cells in fibrotic and cancerous tissues of areca nut chewers, with HSP27/HSP47 upregulation potentially associated with COX-2 activation [11].

Thus, long-term areca nut chewing drives oral carcinogenesis through a synergistic interplay of chemical components and persistent physical irritation. Together, they induce and sustain oxidative stress in the oral mucosa, leading to ROS accumulation and disruption of the antioxidant system. This persistent oxidative stress activates inflammatory signaling pathways such as NF-κB, creating a self-reinforcing “oxidative stress-inflammation” cycle. While short-term compensatory mechanisms like the SIRT3/HO-1 pathway can initially mitigate damage, prolonged exposure eventually leads to their failure, converting protective responses into pro-carcinogenic processes. Consequently, a chronic microenvironment characterized by sustained oxidative stress, inflammation, DNA damage, and fibrosis is established. This environment continuously promotes DNA damage and mutation, while also enhancing proliferation, inhibiting apoptosis, and facilitating invasion. Collectively, these multi-stage cascades drive the progression of oral mucosa from chronic injury to precancerous lesions (e.g. OL and OSF) and ultimately to OSCC.

#### TGF-β activation and collagen dysregulation induced by areca nut in oral cancer

**Figure 5.**
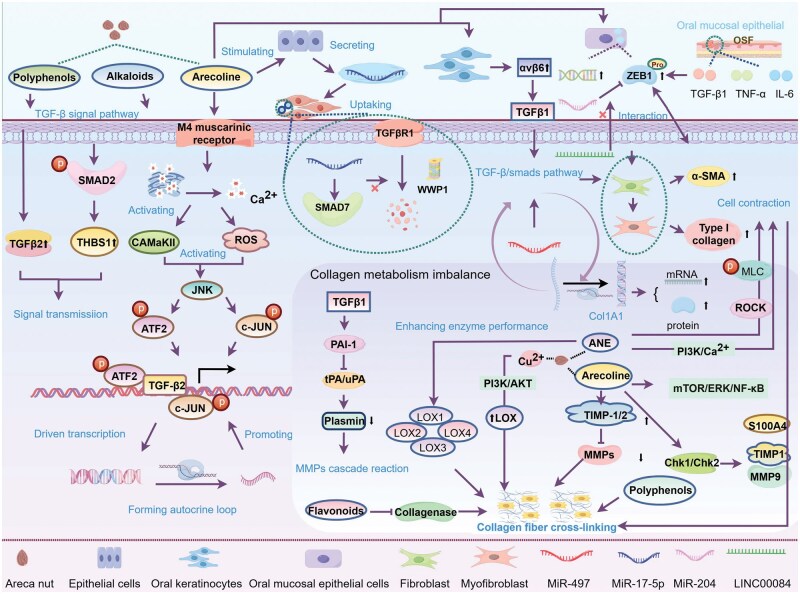
Molecular mechanism of areca nut-mediated TGF-β activation and collagen dysregulation. Areca nut polyphenols and alkaloids directly activate TGF-β/SMAD pathway, promoting SMAD2 phosphorylation and pro-fibrotic factor THBS1 (thrombospondin-1) expression. Arecoline triggers intracellular Ca^2+^ release and ROS generation via M4 muscarinic receptor, activating CaMKII–JNK–ATF2/c-Jun axis to upregulate TGF-β2 transcription to form autocrine amplification loop. Meanwhile, arecoline-stimulated miR-17-5p-rich exosomes enhance TGF-β receptor stability by inhibiting Smad7/WWP1. Sustained TGF-β activation induces α-SMA positive myofibroblast transformation. The transcription factor ZEB1 (induced by arecoline and inflammatory factors) directly binds α-SMA promoter, cooperating with the lncRNA LINC00084 (antagonizing miR-204-mediated ZEB1 suppression) to exacerbate this process. In terms of collagen metabolism imbalance, ANE and arecoline promote collagen Col1A1 expression through TGF-β1. High copper ions (Cu^2+^) concentrations activate PI3K/AKT to upregulate LOX family, catalyze collagen cross-linking, and enhance ECM stiffness. Collagen degradation is inhibited by multiple mechanisms: (1) TIMP-1/TIMP-2 inhibit MMP activity; (2) TGF-β1 induces PAI-1 to block the plasmin-MMP activation cascade; (3) areca nut polyphenols directly promote collagen cross-linking to resist enzymatic hydrolysis. ECM remodeling and cell contraction activate the PLC/IP3/Ca^2+^ pathway and Rho/ROCK through ANE, triggering CaMKII/myosin light chain (MLC) phosphorylation to cause abnormal contraction of fibroblasts, forming a positive feedback loop with ECM sclerosis.

Areca nut components activate the TGF-β signaling pathway through multiple mechanisms, inducing fibroblast transdifferentiation into myofibroblasts, a critical step in OSF and oral carcinogenesis (Fig. 5).

Polyphenols and alkaloids in ANE directly activate TGF-β signaling [71], promoting SMAD2 phosphorylation and upregulating TGF-β2 and its downstream target thrombospondin-1. Arecoline acts on the muscarinic acetylcholine receptor (M4 subtype), triggering Ca^2+^ release and activating CaMKII while inducing ROS production. Both ROS and Ca^2+^/CaMKII activate JNK, which promotes ATF2 and c-Jun phosphorylation. Phosphorylated ATF2/c-Jun bind to the TGF-β2 promoter, driving its transcription and forming a sustained autocrine loop [72]. Areca alkaloids also stimulate epithelial cells to secrete exosomal miR-17-5p, which targets Smad7 in fibroblasts, blocking WWP1-mediated TGFBR1 degradation and enhancing TGF-β signaling [47]. Additionally, arecoline upregulates integrin αvβ6 in oral keratinocytes via the M4 receptor, activating latent TGF-β1 and promoting myofibroblast transformation [73]. miR-497 further amplifies TGF-β pathway activity by triggering the TGF-β1/Smads cascade. TGF-β activation induces fibroblast differentiation into α-SMA-positive myofibroblasts and promotes ECM synthesis, particularly type I collagen. miR-497 upregulates type I collagen and α-SMA via TGF-β1/Smads, enhancing myofibroblast contractility, migration, and invasiveness [46].

Zinc finger E-box binding homeobox 1 (ZEB1), a key EMT regulator, is upregulated by arecoline in oral mucosal epithelial cells. Inflammatory (e.g. IL-6, TNF-α) and fibrotic factors (e.g. TGF-β1) in OSF lesions synergistically induce ZEB1, forming an “inflammation–fibrosis” cycle. In myofibroblasts, ZEB1 binds to the α-SMA promoter, activating its transcription and promoting transformation and collagen contraction [74]. The lncRNA LINC00084 is upregulated in areca-treated buccal mucosal fibroblasts and OSF tissues, promoting myofibroblast activation by alleviating miR-204-mediated ZEB1 suppression [75]. Arecoline also binds to PDE4A in fibroblasts, enhancing its activity and reducing cAMP levels; this activates the Epac1 pathway, synergizing with TGF-β to promote α-SMA and Col1A1 expression [76].

Persistent TGF-β activation by areca nut disrupts collagen homeostasis, leading to excessive deposition and structural abnormalities. ANE and arecoline activate TGF-β1, promote procollagen gene transcription, and upregulate collagen-processing enzymes [70]. High copper levels in areca nut activate PI3K/AKT signaling, upregulating LOX, which catalyzes collagen cross-linking and enhances ECM stiffness [64]. Elevated LOX family members further promote collagen cross-linking, forming a feedback loop of “PI3K/AKT activation–tissue hypoxia–pro-carcinogenic microenvironment” [64].

Areca nut inhibits collagen degradation through three mechanisms: (i) upregulating TIMP-1/TIMP-2 to inhibit MMP activity [11, 77]; (ii) directly inhibiting collagenase via flavonoids [70]; (3) inducing TGF-β1 to upregulate PAI-1, which inhibits plasmin generation and MMP activation [70]. Areca polyphenols also increase collagen cross-linking density, reducing collagenase susceptibility [11].

Areca nut extract induces oral fibroblast contraction by activating the PLC/IP3/Ca^2+^ pathway: IP3 triggers Ca^2+^ release, activating CaMKII and promoting myosin light chain phosphorylation to drive contraction. ANE also enhances MLC phosphorylation via Rho kinase, inhibitable by HA1077 [78]. Abnormal contraction promotes collagen secretion and ECM stiffening. Additional synergistic pathways include: (i) areca alkaloids upregulate S100A4 via mTOR/ERK/NF-κB, regulating TIMP1 and MMP-9 and exacerbating collagen gel contraction [60]; (ii) areca components activate Chk1/Chk2, regulating cell cycle, apoptosis, and MMP-9/TIMP expression to promote carcinogenesis [79].

In summary, areca nut disrupts collagen metabolism by promoting synthesis, inhibiting degradation, and enhancing cross-linking, while driving fibroblast contraction and activating multiple signaling cascades (e.g. TGF-β, PI3K/AKT, Rho/ROCK), collectively promoting OSF and oral cancer.

#### Areca nut-mediated immune microenvironment imbalance drives OSF–OSCC transformation

The tumor immune microenvironment forms the functional core of the TME. It is dynamically composed of infiltrating immune cells, cytokine/chemokine networks, and immunoregulatory molecules. This system plays a decisive role in maintaining the balance between immune surveillance and immune escape [80, 81]. Existing studies have confirmed that the tumor immune microenvironment plays a key regulatory role in various solid tumors, such as lung cancer and renal cell carcinoma. It influences tumor initiation, progression, malignant evolution, and therapeutic resistance. These effects are achieved through multi-layered and highly complex mechanisms [82–86]. For instance, tumor cells can drive metabolic reprogramming involves sugars, lipids, amino acids, and other metabolites via epigenetic modifications, such as DNA methylation and histone modifications. This reprogramming not only helps tumor cells adapt to microenvironmental pressures but also directly shapes the functional states of immune cells. These findings make metabolic reprogramming a novel target for immunotherapy [87].

Meanwhile, the host microbiota serves as a critical external regulator of the tumor immune microenvironment. Dysbiosis in its composition and function can profoundly influence anti-tumor immune responses through the immune–oncology–microbiome axis. Moreover, microbiota-based interventional strategies, such as fecal microbiota transplantation and engineered bacteria, are becoming cutting-edge approaches to enhance the efficacy of immunotherapies [88].

**Figure 6.**
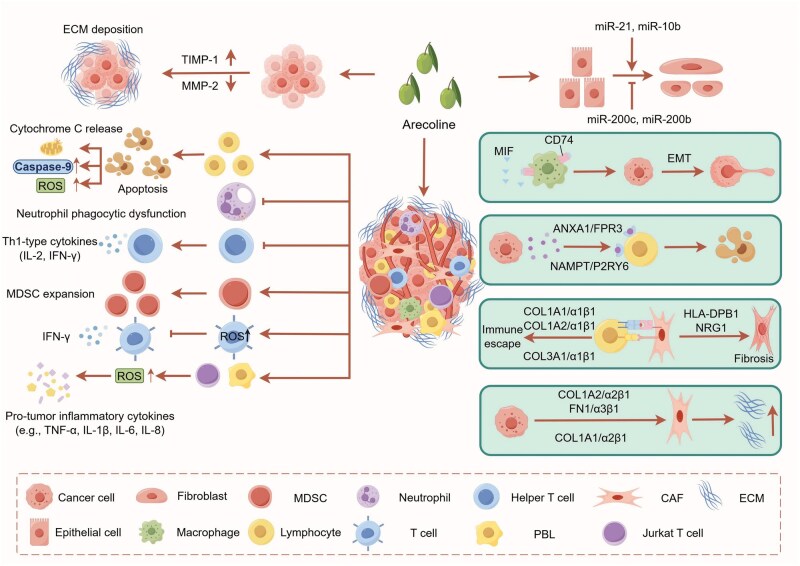
Multi-stage progression model from normal mucosa to invasive oral cancer. Complete progression from normal oral mucosa (basal/prickle/superficial cells) through precancerous lesions (oral lichen planus, leukoplakia, submucous fibrosis, erythroplakia) to invasive oral cancer. Includes stage-specific cellular characteristics, key mechanisms, invasion, and metastasis patterns.

During OSF malignant transformation to OSCC, areca nut-induced TME remodeling plays a central role (Fig. 6). The TME comprises cellular components (tumor cells, activated fibroblasts/myofibroblasts, immune cells), remodeled ECM, and dysregulated intercellular signaling. Areca nut bioactive components (e.g. arecoline) modulate the TME through dual mechanisms: inducing fibroblast transdifferentiation into myofibroblasts to promote excessive collagen synthesis and abnormal ECM deposition; and suppressing immune cell anti-tumor functions to establish an immunosuppressive microenvironment facilitating immune escape. This remodeled TME provides physical and biochemical support for tumor proliferation and invasion while evading immune surveillance, collectively accelerating OSCC development.

First, ANE induces EMT primarily by upregulating transcription factors (e.g. ZEB1/2, Twist, Slug), driving oral fibrosis and carcinogenesis [74, 89, 90]. These factors regulate myofibroblast activation and collagen deposition, and interact with miRNAs to modulate fibrosis. Anti-fibrotic miR-200 family members (miR-200c, miR-200b) target ZEB1/ZEB2 and Slug, downregulating α-SMA and vimentin to inhibit myofibroblast activation [49, 50]. Arecoline suppresses miR-200c/miR-200b, relieving their inhibition on pro-fibrotic molecules and promoting myofibroblast activation. Conversely, miR-10b exerts pro-fibrotic effects by upregulating Twist to enhance myofibroblast activation and collagen contraction; its expression correlates with OSF severity, and its inhibition blocks Twist-mediated fibrosis [51]. Persistent activation disrupts ECM homeostasis, leading to pathological fibrosis [91, 92]. Arecoline also stimulates ECM accumulation by increasing TIMP-1 and inhibiting MMP-2 in fibroblasts [77]. Excessive ECM deposition not only exacerbates fibrosis but also creates a permissive microenvironment for OSCC initiation and progression. Second, areca nut constructs an immunosuppressive and pro-inflammatory TME in OSCC. ANE directly induces mitochondrial apoptosis in lymphocytes via ROS elevation and caspase-9 activation, reducing lymphocyte counts [93]. It also impairs neutrophil phagocytosis [94, 95], suppresses Th1 cytokines (IL-2, IFN-γ), and expands CD11b^+^Gr-1^+^ myeloid-derived suppressor cells (MDSCs) in mice [96]. In T cells, ANE elevates ROS to inhibit activation and IFN-γ secretion, while increasing pro-carcinogenic cytokines (TNF-α, IL-1β, IL-6, IL-8) [97].

Spatial transcriptomics (ST) has revealed TME heterogeneity and cell–cell interactions in OSF-derived OSCC [98]. Immune cells (e.g. T cells, macrophages) form spatial clusters in stromal/tumor regions and interact extensively with tumor cells and CAFs. Key interactions include: CD74-MIF mediating macrophage–epithelial crosstalk to induce EMT and impair immunity [99, 100]; malignant epithelial cells expressing immunosuppressive ligands (e.g. ANXA1/FPR3, NAMPT/P2RY6) [101–103]; and HLA-DPB1 on immune cells binding NRG1 on CAF1, potentially influencing EMT and fibrosis [104, 105]. CAF1 further interacts with immune cells via collagen/integrin pairs (COL1A1/α1β1, COL1A2/α1β1, COL3A1/α1β1), forming a physical barrier that restricts immune cell infiltration [106, 107]. CAF1 also interacts with epithelial cells through COL1A1/α2β1, COL1A2/α2β1, and FN1/α3β1, reinforcing the barrier and promoting immune escape [108]. CAF1 likely originates from epithelial cells that underwent complete EMT, localizing at the tumor–stroma interface with strong ECM remodeling capacity; CAF2 comprises myofibroblast-like cells in stromal/OSF areas. Both interact with immune cells via uncharacterized ligand–receptor pairs, collectively shaping the immunosuppressive microenvironment [107, 108]. These findings suggest potential strategies for preventing OSF malignant transformation and treating OSF-derived OSCC (Table 1).

**Table 1.** Key molecules and specific mechanisms of arecoline-induced oral cancer.

Key nodes	Biological process	Core factors/markers	Reference(s)
DNA damage–repair dysfunction	Induce DNA double-strand breaks; downregulate mismatch repair genes (*MLH1*, *MSH2*); rendering the damage unable to be properly repaired.	DNA double-strand break markers: γ-H2AX, 8-OHdG; downregulated expression of *MLH1*/*MSH2*.	[40, 49, 59, 109, 110]
High-frequency mutations in driver genes and pathway selection	Activation and amplification of oncogenes, coupled with inactivation of tumor suppressor genes, lead to uncontrolled proliferation and survival of cells.	High-frequency mutation:*TP53* (76%–80%), *CDKN2A*, *PIK3CA*, *HRAS*, *EGFR*.	[24–27, 29, 30]
Epigenetic and non-coding RNA network dysregulation	Extensive regulation of cancer-related pathways disrupts non-coding RNA networks, affecting the expression of tumor suppressor genes and oncogenes.	Oncogenic molecule (miR-497, miR-17-5p, miR-10b); tumor suppressor molecule(miR-34a, miR-200b/c), lncRNA: LUCAT1, MIR31HG, HIF1A-AS1.	[39, 51, 52, 111, 112]
Hub role of the TGF-β signaling pathway	Inducing EMT and cancer-associated fibroblast activation.	TGF-β1/2, p-SMAD2/3, SMAD7; ZEB1, α-SMA, COL1A1; E-cadherin, N-cadherin, Vimentin.	[42, 46, 47, 49, 50, 75]
Oxidative stress–inflammation axis and homeostasis disruption	ROS surge, activating inflammatory pathways; dysfunction of endogenous antioxidant defense systems.	Source of oxidative stress: ROS/RNS; critical medium: COX-2/PGE2, NF-κB; SIRT3/FOXO3A/SOD2 activity conversion.	[51, 52, 58, 59, 67]
Metabolism and proteome reprogramming	Adaptation to proliferation and oxidative stress, synthesizing large amounts of ECM and stress proteins.	Disorders in glutathione metabolism; overexpression of heat shock proteins (HSP27, HSP47); abnormal expression of cell keratin 17.	[11, 52, 58–60]
Immunosuppressive microenvironment	The function of effector immune cells is suppressed, while the subpopulations of suppressor immune cells are expanded, and immune checkpoints are activated.	Immunosuppressive cells: Tregs, M2 tumor-associated macrophages, MDSCs; immunosuppressive signal: PD-L1, TGF-β, IL-10.	[31–40, 47, 96, 113]
Heterogeneous activation of cancer-associated fibroblasts (CAFs)	CAFs are the primary builders and regulators of the TME, with different functional subpopulations collaboratively promoting fibrosis, immune evasion, and tumor progression.	Markers: α-SMA, FAP. Functional subpopulations: CAF1 (ECM remodeling type), CAF2 (contractile type).	[46, 74, 104–107]
Multimodal synergistic carcinogenic effects of areca nut	Chemical toxicity, physical damage, and biochemical environmental changes synergistically interact, with tobacco (benzo[a]pyrene-DNA adducts), alcohol (acetaldehyde), high-risk HPV (E6/E7 oncogenes), and areca nut producing additive or synergistic effects.	Chemical: Arecoline, arecolamine. Physical: Coarse fiber causes microtrauma. Biochemical: Localized hyper pH, copper ion release.	[2, 17, 29, 30, 66, 67]

In summary, areca nut drives the malignant transformation of OSF to OSCC through a dual mechanism. On one hand, its active components (e.g. arecoline) upregulate transcription factors such as ZEB1/2 and Twist. They also inhibit anti-fibrotic miRNAs while promoting pro-fibrotic miRNAs. This induces EMT, myofibroblast activation, and abnormal ECM deposition. Together, these changes provide both a physical scaffold and a biochemical foundation for carcinogenesis. On the other hand, areca nut establishes an immunosuppressive microenvironment. It induces lymphocyte apoptosis, impairs neutrophil and T-cell functions, expanding MDSCs. It also leverages spatially specific cellular interactions include CD74-MIF signaling and collagen/integrin pairing. These interactions form a physical barrier and an immunosuppressive signaling network. Collectively, all these actions promote immune escape and tumor progression.

## Specific biomarkers for areca nut-associated oral cancer: from etiological drivers and risk early-warning to precision diagnosis and treatment

### Risk prediction and biomarkers for early diagnosis

#### Multidimensional determinants of early-stage risk in areca nut-associated oral cancer

**Figure 7.**
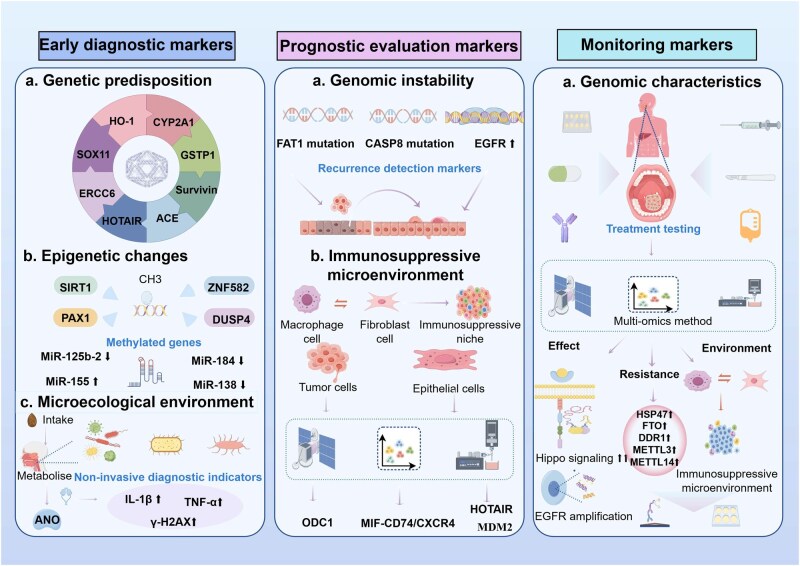
Early diagnostic, prognostic evaluation, and monitoring markers for areca nut‑associated oral cancer. Early diagnostic markers include: (a) genetic susceptibility genes (e.g., *SOX11*, *HO1*, *ACE*, *GSTP1*, etc.); (b) epigenetic alterations (methylation of *SIRT1*, *PAX1*, *ZNF582*); (c) oral microecological dysbiosis (pathogen intake). Prognostic evaluation markers include: (a) genomic instability (*FAT1*/*CASP8* mutations, *EGFR* amplification); (b) immunosuppressive microenvironment (tumor‑associated macrophages, cancer‑associated fibroblasts, and immunosuppressive niches). Treatment monitoring markers involve: (a) genomic features and (b) immunosuppressive microenvironment‑related molecules.

The risk for early-stage areca nut-associated oral cancer arises from a confluence of genetic predisposition, direct carcinogen insult, and oral microbial dysbiosis. Genetic susceptibility constitutes a fundamental intrinsic determinant, characterized by significant gene–environment interactions wherein polymorphisms in multiple genes modulate individual risk in the context of areca nut chewing (Fig. 7). For instance, longer (GT)*n* repeat alleles within the *HO-1* gene promoter region are significantly associated with elevated OSCC risk, with a pronounced effect in buccal mucosa carcinomas [114]. The DD genotype of the *ACE* gene I/D polymorphism serves as an independent risk factor for oral precancerous lesions, particularly among non-smokers [115]. Variants in genes such as *CYP26A1* and the AA genotype of *GSTP1* further augment disease risk. The *GSTP1* AA genotype compromises detoxification capacity by inhibiting c-Jun phosphorylation and pro-apoptotic gene expression, leading to elevated levels of the oxidative DNA damage marker 8-OHdG [109, 110]. Additionally, single nucleotide polymorphisms (SNPs) in genes including *Survivin*, HOTAIR, and CDKN2B-AS1 demonstrate significant interaction with areca nut chewing, collectively contributing to a substantially increased OSCC risk. Variants in *SOX11*, *CYP26B1*, *AURKA*, *MIR155HG*, and *RAGE* have also been identified to synergistically elevate OSCC risk in combination with areca nut exposure [116–125].

Epigenetic regulation represents another critical layer, with its associated molecular alterations offering promising targets for early detection. Regarding DNA methylation abnormalities, research by Islam et al. demonstrated that SIRT1 methylation levels are significantly higher in OSCC tissues from areca nut chewers compared to both non-chewer OSCC patients and healthy controls. *In vitro* studies confirmed that areca nut exposure induces *SIRT1* hypermethylation, resulting in transcriptional downregulation and reduced protein expression. Notably, healthy areca nut chewers also exhibit significantly elevated *SIRT1* methylation in buccal mucosal scrapings relative to non-chewers, with a positive correlation between chewing duration and methylation level. These findings implicate *SIRT1* DNA hypermethylation in areca nut-driven oral carcinogenesis and highlight its potential as an early biomarker for predicting malignant transformation [126]. Adhikari et al. reported promoter hypermethylation and consequent mRNA downregulation of Dual Specificity Phosphatase 4 (*DUSP4*) in areca nut-treated human gingival epithelial progenitor cells, an effect that persisted following long-term exposure. Clinical sample analysis revealed that *DUSP4* methylation levels were significantly higher in OSCC tissues from areca nut chewers than in those from non-chewers or healthy controls, indicating *DUSP4* hypermethylation as a specific molecular event in areca nut-associated oral carcinogenesis and its potential utility for early risk prediction [127]. Cheng et al. using quantitative methylation-specific PCR on oral scrapings, evaluated five genes across cohorts of normal mucosa, oral precancerous lesions, and OSCC patients. They identified *ZNF582* and *PAX1* methylation (*ZNF582*m, *PAX1*m) as effective biomarkers, with methylation levels and positive rates escalating with histopathological severity. Importantly, areca nut chewing alone or in combination with smoking/alcohol use was associated with hypermethylation of these genes. A significant decrease in *ZNF582*m and *PAX1*m levels was observed post-treatment in OSCC patients, underscoring the diagnostic value and clinical applicability of these methylation markers in non-invasive scrapings [128].

Dysregulation of non-coding RNA expression is also a key component of the epigenetic landscape in this malignancy. Studies associate areca nut chewing with significant upregulation of miR-155 and downregulation of miR-125b-2, miR-138, and miR-184, among others. These aberrant miRNA expressions likely function in early carcinogenic stages, providing a molecular foundation for non-invasive liquid biopsy and risk stratification [129]. The aforementioned genetic variations in lncRNAs like HOTAIR and CDKN2B-AS1 and their interactions with areca nut further elaborate the role of epigenetic networks in oral cancer development.

Direct damage from areca nut constituents is central to its carcinogenicity. Tsai et al. found that arecoline suppresses DNA repair by downregulating p53 expression. Its *in vivo* metabolite, arecoline N-oxide (ANO), was identified as a more potent ultimate carcinogen [50]. Crucially, in animal models, ANO induced a significantly higher incidence of oral squamous hyperplasia, leukoplakia, and OSCC. Mechanistically, ANO activates the NOTCH1/FAT1 signaling axis, upregulates proliferation markers (PCNA, Ki67), induces pro-inflammatory cytokines (IL-1β, TNF-α), and causes DNA damage (γ-H2AX), thereby driving epithelial malignant transformation [59].

Furthermore, oral microbial dysbiosis presents as an independent early-warning indicator. Areca nut chewing elevates the abundance of pathogenic bacteria such as *Fusobacterium nucleatum* and *Porphyromonas gingivalis*. This shift promotes a chronic inflammatory state conducive to carcinogenesis, and monitoring microbial dynamics offers a novel avenue for early, non-invasive diagnosis [130].

#### Multi-omics strategies for deconvoluting early risk in areca nut-associated oral cancer

Multi-omics analyses have systematically delineated the molecular biomarker signatures specific to areca nut exposure, thereby laying a solid foundation for precise risk stratification and early clinical diagnosis. Integrated analyses of clinical cohorts from the UK (24 OSCC cases, 7 normal controls) and Sri Lanka (27 OSCC cases, 4 normal controls) revealed that, in contrast to smoking/alcohol-related OSCC, areca nut-associated OSCC is characterized by persistent hyperactivation of cell-mediated immune response genes in both tumor tissues and their adjacent non-tumor counterparts. Conversely, genes implicated in tumor invasion and metastasis (e.g. *MMP3*, *PTGS2*) exhibit relatively attenuated expression [131]. This distinct immune-invasion expression profile provides a critical molecular basis for etiological differentiation of OSCC subtypes. Further bioinformatics analyses have identified key genes (e.g. *TAGLN2*, *CCND2*, *CCL8*) that undergo dysregulation during the early stages of areca nut-driven carcinogenesis [132]. Additionally, single-cell RNA sequencing studies using an arecoline-induced murine model of oral cancer demonstrated that as tumorigenesis progresses, the proportions of stem cell-like and keratinocyte-like epithelial cell subpopulations are markedly expanded. The gene expression signatures of these subpopulations are significantly enriched in myelocytomatosis oncogene (MYC) target pathways, highlighting potential candidate biomarkers for ultra-early detection of oral precancerous lesions [133]. The dynamic evolution of these specific cell states and their core pathways provides novel targets for ultra-early risk identification at the precancerous stage.

### Prognostic assessment and molecular subtyping biomarkers

Areca nut-associated OSCC exhibits significant clinical heterogeneity. Therefore, accurate prognostic assessment requires integrating multidimensional information. This includes genomics, the TME, and key molecular pathways.

Genomic instability serves as a central prognostic driver of tumor evolution. Whole-genome sequencing studies have delineated a distinct driver gene profile in areca nut-associated OSCC. Characteristic events include *FAT1* inactivation mutations, high-frequency *CASP8* mutations, and *EGFR* gene amplification, all associated with enhanced tumor aggressiveness and increased recurrence risk [134, 135]. Furthermore, elevated *TRPM8* expression has been identified as a reliable indicator of lymph node metastasis and shorter disease-free survival [136].

By integrating multiple GEO datasets (GSE215403, GSE208253, GSE220978), the TCGA HNSC dataset, and a clinical cohort from Qilu Hospital with single-cell and spatial transcriptomic analyses, Zhao et al. revealed a key mechanism in OSCC derived from OSF. They found that INHBA^+^ macrophages and pro-inflammatory cancer-associated fibroblasts (iCAFs) work together through the INHBA signaling axis. This synergy promotes Treg differentiation, fostering an immunosuppressive niche. This niche is associated with poor immunotherapy response in this OSCC subtype [137]. The same study also showed that an evaluation system based on stroma-derived TGFBI and endothelium-expressed HYAL1, independently correlates with patient survival. This provides an objective tool for prognostic assessment [138].

In another study, Zhi et al. combined ST and spatial metabolomics data. The datasets covered 4 clinical OSF-derived OSCC samples, the public scRNA-seq database GSE195832, the TCGA-OSCC cohort (*n *= 394), and microarray data from GSE37991. Their work clarified the intratumoral heterogeneity in OSF-derived OSCC and proposed a malignant progression model centered on an “ISC-pEMT-CAF-like phenotype.” Furthermore, they identified a key enzyme in polyamine metabolism—ornithine decarboxylase 1, which is specifically enriched in tumor regions. Its activity directly correlates with tumor proliferation and progression, marking it as a potential prognostic biomarker and therapeutic target [139].

A separate scRNA-seq analysis included tissues from 19 OSF patients, six healthy controls, and blood samples from four individuals. The analysis indicated that epithelial cells promote the formation of an immune-tolerant microenvironment. They achieve this through abnormal communication with immune cells via the MIF-CD74/CXCR4 signaling axis [140]. Huang et al. conducted a cohort of 351 male OSCC patients in Taiwan, China and 1272 general population controls. Using MALDI–TOF mass spectrometry and sequencing, they demonstrated that multiple factors collectively contribute to an aggressive phenotype with poor prognosis. These factors include the coexistence of the MDM2 SNP309 G allele with TP53 mutation, HOTAIR polymorphisms linked to EMT, high IL-1β expression, and polymorphisms in HIF-1α and pre-miR-146a [135, 141–143].

### Biomarkers for treatment response prediction and monitoring

Predicting efficacy and dynamically monitoring treatment response are central to personalized management of areca nut-associated OSCC. Identifying molecular correlates of sensitivity and resistance provides a rational basis for therapeutic strategy.

Genomic features offer predictive insights for targeted therapy. EGFR gene amplification at 7p11.2 has been shown to directly predict tumor response to EGFR inhibitors, informing their targeted use [133]. Multi-omics analyses reveal frequent dysregulation of the Hippo signaling pathway in areca nut-associated oral cancers, suggesting a rationale for exploring combination therapies involving PI3K/mTOR and EGFR inhibitors [134].

Chemoresistance involves a multi-layered molecular network. Single-cell transcriptomics implicate activation of the MYC_targets_v1 pathway within specific arecoline-exposed epithelial subpopulations in cisplatin resistance [133]. Epigenetically, upregulation of the m6A methyltransferases METTL3/METTL4 enhances chemoresistance by stabilizing oncogenic transcripts, including MYC [144]. Additionally, aberrant high expression of HSP47, FTO, and DDR1 contributes to a complex resistance phenotype [145–147]. These markers can be accessed via qRT–PCR for mRNA or IHC/ELISA for protein expression.

Tumor microenvironment characteristics critically influence immunotherapy outcomes. In OSF-derived OSCC, the immunosuppressive niche formed by INHBA^+^ macrophages and iCAFs is linked to T-cell exhaustion and primary resistance to PD-1/PD-L1 checkpoint inhibitors [113]. Immunohistochemical assessment of relevant immune cell markers can aid in predicting immunotherapy responsiveness.

Liquid biopsy enables minimally invasive dynamic monitoring of treatment response. Integrated multi-parameter models tracking gene mutations, DNA methylation, and gene expression allow for high-sensitivity detection of molecular residual disease. Clinical studies demonstrate that post-treatment dynamics of *ZNF582* and *PAX1* methylation levels closely mirror disease status, with their elevation detectable months prior to clinical recurrence, thereby creating a critical window for preemptive intervention [148]. Methylation-specific PCR is particularly suited for the sensitive and rapid detection of such epigenetic markers.

## Multi-stage cascading effects of areca nut-induced oral cancer and targeted therapeutic strategies

**Figure 8.**
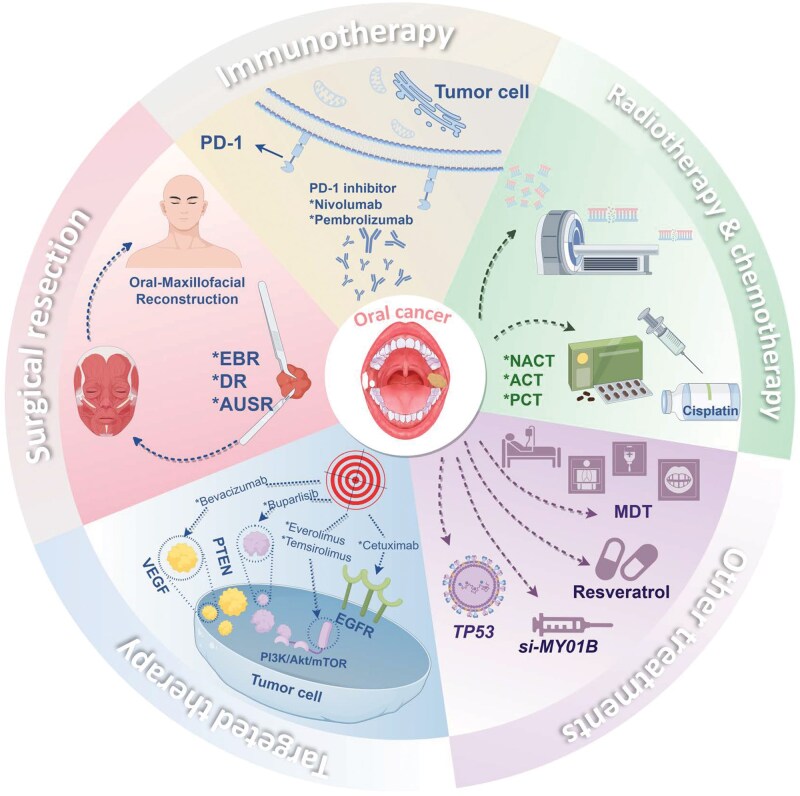
Multi-dimensional diagnosis and treatment strategy system for oral cancer. (i) Local therapy: Surgical resection (EBR: en bloc resection, DR: discontinuous resection, AUSR: anatomical unit resection) and maxillofacial reconstruction; (ii) Systemic therapy: Chemoradiation synergy (NACT: neoadjuvant, ACT: adjuvant, PCT: palliative, chemotherapy drugs: cisplatin; radiotherapy techniques), immunotherapy (PD-1 inhibitors nivolumab/pembrolizumab), targeted therapy (VEGF, EGFR, and PI3K/Akt/mTOR interventions), which simultaneously incorporates multidisciplinary collaboration (MDT). (iii) Emerging modalities: gene therapy (e.g. TP53 function repair, si-MYO1B), and natural compounds (resveratrol applications). Comprehensive coverage spanning local control to systemic regulation, traditional to cutting-edge strategies.

Current clinical management of oral cancer has established a comprehensive model centered on surgical resection, with multidisciplinary team collaboration. For early-stage patients, radical surgery is the primary approach, with growing focus on preserving function and morphology. For locally advanced or high-risk cases, postoperative adjuvant radiotherapy and chemotherapy are standard [104, 105, 149–152]. In systemic therapy, cisplatin-based chemotherapy remains the cornerstone, while targeted drugs and immunotherapy offer new options for recurrent/metastatic patients [153–157] (Fig. 8).

However, these conventional strategies face challenges in areca nut-induced oral cancer, specifically its high invasiveness and recurrence propensity. This requires optimizing and expanding existing treatments based on deeper understanding of its pathogenic mechanisms. The carcinogenic effect of areca nut is a multi-step, multi-level process, its bioactive components induce genomic instability, disrupt the epigenetic regulatory network, and ultimately dysregulate key protein functions and signaling pathways, driving OSF malignant transformation to OSCC. Based on these mechanisms, precise therapies for areca nut-associated oral cancer can be developed.

### Gene repair: targeting genomic instability and DNA damage response

In genetic background, the KIR2DL1-HLA-C2 genotype and MMP-1 2G allele are associated with young OSCC patients. *TP53* is the most frequently mutated gene in OSCC; while its mutation frequency is lower in young non-smoking tongue cancer patients than in young smokers and the general OSCC population, mutation types and p53 protein expression differ [158]. Additionally, promoter CpG island hypermethylation of *RASSF1A*, *RASSF2A*, *MGMT*, *DAPK*, and *FHIT* is detectable in early OSCC [158], providing directions for future gene editing to repair mutations or restore gene function.

Studies show arecoline induces widespread abnormal gene expression in oral mucosal cells, with *MYO1B* identified as a key gene in arecoline-related oral cancer. RNA interference-mediated *MYO1B* suppression significantly reduced proliferation, clonogenicity, migration, and invasion of arecoline-transformed oral cells (DOK/trans) and OSCC cells (e.g. SAS), indicating targeting MYO1B can reverse malignant phenotypes [159]. At the DNA damage level, areca nut components cause direct or ROS-induced indirect DNA damage, activating intracellular DNA damage checkpoint proteins Chk1 and Chk2. Chronic, sustained exposure leads to aberrant cell cycle arrest and apoptosis dysregulation, laying the groundwork for malignant transformation [75, 160]. Targeting this mechanism, Chk1/Chk2 inhibitors (e.g. AZD7762) can restore normal cell cycle progression and eliminate cells with severe DNA damage, preventing carcinogenesis initiation.

### Epigenetic regulation: targeting widespread dysregulation of non-coding RNA networks

Areca nut drives ECM remodeling and accelerates tumor cell metastasis/invasion by regulating non-coding RNA expression networks, thereby disrupting downstream signaling pathways.

In areca nut-associated oral cancer, miRNAs play a central regulatory role. Studies have identified 39 oncogenic and 45 tumor-suppressive miRNAs involved in this process [161]. Specifically, key miRNAs (Let-7c, miR-130a-3p, miR-361-5p, miR-99a-5p, miR-29c-3p, let-7d-5p) show characteristic dysregulation in OSCC, influencing malignant phenotypes (proliferation, apoptosis, differentiation, migration) and exhibiting specific expression profiles in aggressive tongue cancer of young patients [162].

Arecoline disrupts miRNA network balance via multiple mechanisms. First, it reduces expression of critical tumor-suppressive miRNAs, most notably miR-145. Long-term arecoline exposure markedly lowers miR-145 in oral epithelial cells, losing its targeted inhibition on stemness transcription factors Oct4/Sox2. This promotes cancer stem cell properties, EMT, and chemoresistance [111]. Arecoline also suppresses other tumor-suppressive miRNAs (miR-22, miR-886-3p [163]) and downregulates miR-34a, miR-200b/c, miR-499a-5p, which normally inhibit oncogenic proteins (Axl, ZEB1/ZEB2) and enhance chemosensitivity [41, 46, 161].

Second, arecoline induces abnormal overexpression of pro-oncogenic miRNAs. It disrupts epithelial–fibroblast communication, upregulating epithelial-derived miR-17-5p to mediate fibroblast differentiation via the TGF-β pathway. Arecoline also overexpresses miR-21, miR-10b, miR-497, activating pro-fibrotic/pro-carcinogenic pathways (e.g. TGF-β) to drive fibroblast-to-myofibroblast transdifferentiation, ECM deposition, and cell invasion [39].

Precision interventions targeting miRNA dysregulation have been developed. For abnormally upregulated miRNAs (e.g. miR-17-5p, miR-21), siRNA or CRISPR-dCas9 can specifically knock down their expression to dismantle pro-carcinogenic networks. For downregulated tumor-suppressive miRNAs, replacement strategies include: delivering miR-34a mimics via nanocarriers to inhibit the Axl/Akt pathway, delivering miR-200b/c to suppress ZEB1/ZEB2 and reverse EMT, or delivering miR-499a-5p to restore tumor suppression. For miR-145 downregulation, multi-level interventions include: targeted delivery of miR-145 mimics, developing small-molecule inhibitors against Oct4/Sox2, or using miR-145 as a biomarker for personalized therapy [111].

Beyond miRNAs, other non-coding RNAs contribute to areca nut-associated oral cancer pathogenesis. lncRNAs form a key regulatory layer: areca nut treatment dysregulates lncRNAs (HIF1A-AS1, MIR31HG, MIAT). These act as “molecular sponges” (e.g. MIAT adsorbing miR-342-3p) or directly regulate gene expression to promote cellular stress response, migration, invasion, and anti-apoptosis. Notably, arecoline upregulates GAS5, activating downstream pathways to induce abnormal myofibroblast activation [112]. Targeting these oncogenic lncRNAs—using siRNA or CRISPR-dCas9 to knock down HIF1A-AS1 (inhibit migration), MIR31HG (weaken invasion), or GAS5 that effectively interferes with their pro-carcinogenic effects. tRNA-derived small RNAs also participate: tiRNA-Val-CAC-002 is dose-dependently upregulated by arecoline. It mediates ITGB3 (integrin beta 3) expression, regulates the PI3K/AKT pathway, activates fibroblast autophagy, and promotes abnormal expression of fibrosis-related proteins (collagen I/III, α-SMA), driving OSF [164]. Targeting this mechanism includes: using specific inhibitors to suppress tiRNA-Val-CAC-002 overexpression; restoring ITGB3 via overexpression vectors; or using autophagy inhibitors (e.g. chloroquine) to block excessive autophagy, delaying OSF progression to oral cancer [58].

This multi-layered non-coding RNA regulatory network deepens understanding of arecoline’s carcinogenic mechanisms and provides abundant potential targets for precise therapies.

### Molecular-targeted therapy: from signaling pathway intervention to functional protein regulation

Arecoline drives the initiation and progression of oral cancer by disrupting multi-level molecular networks. At the molecular level, intervention strategies primarily focus on two core directions: (i) targeting abnormally activated oncogenic signaling pathways; (ii) correcting the metabolic and expression imbalances of key functional proteins.

In the context of signaling pathway-targeted therapy, specific small-molecule inhibitors can be used to precisely block core pathways activated by arecoline. For instance, ERK inhibitors (e.g. PD98059), TGF-β receptor inhibitors (e.g. Galunisertib), or Akt inhibitors (e.g. MK-2206) can respectively inhibit cell proliferation, excessive ECM synthesis, and cell survival signals [165]. For the newly identified “Egr-1–Wnt5a” regulatory axis, Egr inhibitors (e.g. MMA) or Wnt5a neutralizing antibodies can effectively block abnormal proliferation induced by low-dose arecoline [166]. Furthermore, targeting the pentose phosphate pathway activation driven by the c-MYC/NRF2 metabolic axis can be achieved either by combining G6PD inhibitors (e.g., DHEA) with standard chemotherapeutics (e.g., cisplatin) or by directly targeting NRF2 itself. This approach effectively reverses tumor metabolic reprogramming and inhibits malignant progression [163].

Regarding functional protein network regulation, strategies focus on correcting dysregulation of metabolism-, matrix-, and stress-related proteins. To target arecoline’s metabolic detoxification process, antioxidants such as *N*-acetylcysteine and quercetin can scavenge ROS and promote detoxification [167]. For AKR1B10, a metabolic enzyme upregulated by arecoline, its specific inhibitor oleanolic acid can effectively reverse AKR1B10 mediated EMT, cancer stemness properties, and chemoresistance [57]. At the ECM level, developing S100A4 inhibitors helps regulate the TIMP1/MMP9 balance and counteract fibrotic processes [168]. Concurrently, applying heat shock protein (HSP) inhibitors (e.g., quercetin) or antioxidants (e.g., EGCG) can weaken the stress defense capacity of cancer cells (mediated by HSPs and other molecules), thereby enhancing their sensitivity to treatment [56, 169, 170].

Systematically targeting key nodes in oncogenic signaling pathways and precisely regulating dysregulated functional proteins constitute the core therapeutic strategy for arecoline-induced oral cancer. This approach lays a solid theoretical foundation for the development of high-efficacy, low-toxicity precision therapies.

## Limitations in areca nut-induced oral cancer research

Although progress has been made in understanding the mechanisms, prevention, and treatment of areca nut induced oral cancer, several deep seated and interconnected limitations remain, creating multiple gaps from basic research to clinical translation.

### Pathogenic mechanism studies rely on oversimplified models

Most research focuses on single components like arecoline in isolated settings. Yet areca nut alkaloid content varies by region and product (2–10 mg/g), making toxicity thresholds product. Commonly used doses (e.g., 0.2–0.6 mg/mL) do not reflect real-world exposure [171–173]. Human chewing combines chronic mechanical friction with multiple chemicals, not single compounds. This gap limits study of synergistic effects and clinical relevance. Recent models, such as one integrating physical damage and chemical stimulation, better mimic real chewing. Still, animal oral anatomy and microbiota differ from humans, and simulations only approximate real conditions [173]. Moreover, most reports of multi-dimensional changes (genetic, epigenetic, microbial) remain correlative [134, 173–175].

### Clinical research lacks generalizable data and tailored treatments

Epidemiological and clinical data are largely from high consumption regions (e.g. Southeast Asia, southern China) [176–182], with a lack of large scale, multi population prospective cohorts. This hinders validation of early-diagnostic markers across populations. In treatment, standard oral cancer protocols are used, ignoring the unique microenvironment of areca nut-related cases-marked by severe fibrosis (OSF). This fibrotic, immunosuppressive setting raises surgical difficulty, lowers chemo-radiotherapy sensitivity, and complicates targeted or immunotherapy development, which lacks strong clinical evidence.

### Public health prevention faces cultural and technical barriers

Areca nut use is often deeply embedded in local economies and social customs [174, 183–186], resisting measures like sales restrictions and education. Health campaigns are mostly informational, lacking personalized interventions for addiction. Although some early molecular markers exist, turning them into low-cost, accessible screening tools for primary care remains a hurdle, delaying “early screening and prevention.”

### Cutting edge technologies are still in preliminary stages of application

Emerging tools such as ST and multi omics could reveal spatiotemporal dynamics in areca nut carcinogenesis [187–196], but current use remains superficial. For example, spatial analyses are mostly limited to static sections, lacking dynamic data from precancerous to invasive stages. Similarly, multi omics integration often stays at the level of “correlation listing” without probing causal mechanistic links between microbiome shifts and epithelial genomic instability.

### Technical and integrative limitations in multi-omics methodologies

Current research on areca nut-associated oral carcinogenesis is constrained by fundamental challenges in multi-omics data harmonization and interpretation. Multiple omic data types exist, such as genomic, single-cell transcriptomic, and spatial omic profiles. However, integrating these disparate datasets remains technically challenging. This is primarily due to the lack of robust, standardized analytical pipelines [50, 59, 126–133]. This impedes the systematic reconstruction of cohesive molecular networks essential for a mechanistic understanding of the disease.

In summary, research on areca nut-related oral cancer remains fragmented: basic mechanisms do not match real exposure, data lack causal integration, clinical findings need broader validation, treatments overlook disease-specific traits, and prevention concepts are not implemented. Progress will require more realistic models, deeper causal studies, wider clinical collaboration, better technology use, and pragmatic public-health strategies.

## Conclusions and future perspectives

Areca nut drives oral carcinogenesis through a multilevel pathological cascade, spanning molecular alterations to microenvironmental remodeling. Key mechanisms include: (i) arecoline-induced oxidative stress, which leads to DNA damage and impairs mismatch repair [40–49]; (ii) epigenetic dysregulation coupled with recurrent mutations (e.g. *TP53* and *HRAS*), disrupting cell-cycle control and apoptosis [25–30]; (iii) sustained activation of signaling pathways such as TGF-β/Smad, PI3K/AKT, and NOTCH, promoting EMT, collagen dysmetabolism, and dense ECM deposition [59, 64, 76, 108]; (iv) the establishment of an immunosuppressive microenvironment through macrophage polarization, regulatory T-cell recruitment, and PD-L1 upregulation [31, 41, 44].

Given this multifactorial pathogenesis, a multi-tiered biomarker framework is essential for early intervention and precision oncology. Early detection requires integration of genetic variants, epigenetic alterations, and microbiome signatures [109, 110, 126, 128]. Single-cell multi-omics analyses have revealed an “immune-activated yet invasion-suppressed” pre-malignant phenotype in areca nut-associated oral carcinogenesis [59, 109, 110, 116–121]. From a technical perspective, this requires a multi-assay approach: TaqMan probes for detecting genetic variants, methylation-specific PCR for analyzing epigenetic markers, 16S rRNA sequencing for microbial profiling, and liquid chromatography-mass spectrometry for quantifying arecoline N-oxide. For predicting and monitoring therapeutic efficacy, promising biomarkers include EGFR amplification, Hippo/MYC pathway dysregulation, altered m6A methylation patterns, and the immunosuppressive INHBA^+^ niche-all of which may indicate responses to targeted therapy, chemotherapy, or immunotherapy [135, 139–143]. Multi-omics technology has demonstrated its systematic application value across multiple medical fields, including oncology [189–196] (e.g., pancreatic cancer, non-small cell lung cancer). In studies of areca nut-associated oral cancer, the integrated analysis of genomic, single-cell, and spatial multi-omics data has systematically delineated driver gene profiles, immune microenvironment remodeling, and metabolic reprogramming features [136–140]. These findings provide key molecular evidence for early risk warning (e.g., SIRT1 methylation, genetic polymorphisms, microbial dysbiosi) and prognostic assessment [132–135]. Current research in this field remains in the developmental stage, and further expansion of the depth and breadth of multi-omics technology applications is expected to offer more systematic scientific support for the precise prevention and treatment of areca nut-associated oral cancer [50, 59, 126–143].

Areca nut-related OSCC is characterized by high invasiveness, multicentricity, and a high recurrence rate (with a 5-year recurrence rate of 40%–60%) [1, 12, 16–20] in clinical. Localized lesions are typically treated with surgical resection combined with free flap reconstruction, while cases with trans-compartmental infiltration often require neoadjuvant chemoradiotherapy. Although postoperative radiotherapy can reduce the risk of local recurrence, long-term areca nut chewers exhibit an about 20% reduction in local control rates, which may be attributed to ECM-mediated radioresistance [64, 65]. In terms of systemic therapy, the efficacy of cetuximab combined with radiotherapy is limited due to overactivation of the PI3K/AKT signaling pathway [164]. PD-1 inhibitors achieve response rates of 25%–30% in MSI-H/TMB-H subtypes, but their effectiveness is constrained by the “immune desert” microenvironment induced by ANEs [113]. Multidisciplinary treatment approaches have improved the 5-year survival rate of stage III-IV patients to 35%–45%. However, several challenges persist: (i) current stratification strategies inadequately account for individual variability and exposure-subtype interactions; (ii) primary healthcare settings lack access to precision radiotherapy and targeted therapies; (iii) CRISPR-based TP53 repair technologies and nanocarrier delivery systems remain in the preclinical research stage. Future research should prioritize three key directions: mechanistically, elucidating the spatial interactions between ECM sclerosis and immune suppression, including identifying fibrosis zone-specific immune checkpoints to guide “defibrosis + immune activation” strategies; therapeutically, utilizing dynamic ctDNA monitoring to optimize treatment timing and exploring novel combination therapies such as COX-2 inhibitors with ferroptosis inducers; translationally, accelerating the evaluation of targeted combination therapies and developing wearable sensors for early detection of precancerous lesions.

Early detection and improved therapies are important, but reducing areca nut consumption remains the most fundamental way to lower the disease burden. Since a precision diagnosis and treatment system for areca nut-related oral cancer is still under development, curbing its use is essential for preventing the disease. As a well-established oral cancer risk factor, reducing consumption is a core public health strategy. Achieving this requires a multi-level, systematic, and comprehensive intervention framework [197, 198]. First, at the policy and legislative level, successful tobacco control frameworks such as the MPOWER strategy can serve as a reference. Measures could include legal restrictions on sales and advertising, health taxes, and mandatory prominent health warnings on packaging [199, 200]. Second, at the clinical and community level, it is recommended to incorporate “areca nut use” as a key health-behavior indicator in routine oral exams and medical histories. Brief cessation counseling should be offered to frequent users [201, 202]. Third, at the individual intervention level, structured behavioral–cognitive intervention programs should be developed and promoted for heavily dependent areca nut users, along with exploration of potential pharmacotherapies to assist in cessation.

In summary, areca nut promotes oral carcinogenesis through a multi-level cascade involving molecular damage to microenvironmental remodeling. The identification of key oncogenic nodal factors within this cascade provides critical targets for interception. Early detection requires integrated multi-modal biomarkers, yet clinical translation faces standardization challenges. OSCC treatment is hampered by high recurrence rates and therapeutic resistance, compounded by inadequate molecular-clinical integration and limited healthcare resources. Future efforts should focus on integrating consumption control policies with advanced early screening and precision therapies, forming a comprehensive prevention-management continuum to reduce disease incidence and improve patient outcomes.
